# Deletion of ghrelin prevents aging‐associated obesity and muscle dysfunction without affecting longevity

**DOI:** 10.1111/acel.12618

**Published:** 2017-06-06

**Authors:** Bobby Guillory, Ji‐an Chen, Shivam Patel, Jiaohua Luo, Andres Splenser, Avni Mody, Michael Ding, Shiva Baghaie, Barbara Anderson, Blaga Iankova, Tripti Halder, Yamileth Hernandez, Jose M. Garcia

**Affiliations:** ^1^ Division of Diabetes Endocrinology and Metabolism MCL Center for Translational Research on Inflammatory Diseases Michael E DeBakey Veterans Affairs Medical Center and Baylor College of Medicine Houston TX USA; ^2^ Department of Health Education College of Preventive Medicine Third Military Medical University Chongqing 400038 China; ^3^ Department of Environmental Hygiene College of Preventive Medicine Third Military Medical University Chongqing 400038 China; ^4^ GRECC VA Puget Sound Health Care System and University of Washington Seattle WA USA

**Keywords:** frailty, growth hormone, growth hormone secretagogue receptor, inflammation, Sarcopenia, wasting

## Abstract

During aging, decreases in energy expenditure and locomotor activity lead to body weight and fat gain. Aging is also associated with decreases in muscle strength and endurance leading to functional decline. Here, we show that lifelong deletion of ghrelin prevents development of obesity associated with aging by modulating food intake and energy expenditure. Ghrelin deletion also attenuated the decrease in phosphorylated adenosine monophosphate‐activated protein kinase (pAMPK) and downstream mediators in muscle, and increased the number of type IIa (fatigue resistant, oxidative) muscle fibers, preventing the decline in muscle strength and endurance seen with aging. Longevity was not affected by ghrelin deletion. Treatment of old mice with pharmacologic doses of ghrelin increased food intake, body weight, and muscle strength in both ghrelin wild‐type and knockout mice. These findings highlight the relevance of ghrelin during aging and identify a novel AMPK‐dependent mechanism for ghrelin action in muscle.

**Highlights**

- Ghrelin deletion modulates appetite and metabolism preventing obesity during aging
- Ghrelin deletion prevents aging‐induced decline in muscle strength and endurance
- Ghrelin deletion also attenuated the decrease in pAMPK in muscle seen with aging
- Longevity was not affected by ghrelin deletion

## Introduction

By the year 2050, the number of individuals over the age of 65 will increase to approximately 80 million in the USA alone, representing 20% of the population. Moreover, more than 4 percent of the population will be ≥ 85 years of age (Bureau of the Census 2008). A hallmark of aging is a decrease in muscle function and overall physical performance that often contributes to the loss of independence, increased frailty, poor balance, disability, morbidity, and mortality. In fact, 1 in 10 community‐dwelling people of age 65 or older and 1 in 2 individuals over the age of 85 require assistance with activities of daily living. Muscle function loss is inherently associated with increased risk of sustaining falls, and fall‐related injuries, length of hospital stays, and healthcare costs (Sayer *et al*., 2006; Wickham *et al*., 1989).

Obesity has reached epidemic proportions, and the highest prevalence is found in men and women aged between 65 and 69 (Sulander *et al*., 2004). It is also associated with increased physical disability, morbidity, and mortality in this age group (Villareal & Holloszy, 2004). Obese elderly individuals have decreased muscle performance compared to normal weight elderly individuals, although their muscle mass is typically increased (Baumgartner *et al*., 2004). The mechanisms underlying the development of muscle dysfunction in the setting of preserved muscle mass in obesity remain incompletely understood and are a barrier to the development of therapeutic options for obese elderly individuals (Rolland *et al*., 2004).

Ghrelin, a 28‐amino acid peptide made primarily in the stomach but also in the brain, adipose tissue, and muscle (Gnanapavan *et al*., 2002), is the endogenous ligand for the growth hormone secretagogue receptor (GHSR‐1a) (Kojima *et al*., 1999). It is a potent growth hormone (GH) secretagogue and orexigenic hormone (Kojima *et al*., 1999), and short‐term administration of pharmacologic doses of ghrelin or ghrelin mimetics increases body weight by enhancing appetite and fat deposition (Garcia *et al*., 2008; Garcia & Polvino, 2007; Patterson *et al*., 2013), and possibly by decreasing energy expenditure (Murphy *et al*., 1998). Activation of this pathway also has anabolic effects in muscle; murine and human studies have consistently shown that ghrelin and ghrelin mimetics increase muscle function and prevent muscle wasting in different settings (Chen *et al*., 2015; DeBoer & Marks, 2006; Garcia *et al*., 2008; Porporato *et al*., 2013). Ghrelin also decreases inflammation, a common mechanism implicated in muscle wasting associated with aging, obesity, and other chronic conditions (Chen *et al*., 2015; Dixit *et al*., 2004). In spite of this evidence from studies using pharmacologic doses of ghrelin, genetic deletion of ghrelin has not been associated with anorexia, or changes in body size or body composition in young animals (Davis *et al*., 2012; Pfluger *et al*., 2008; Sun *et al*., 2003; Tschop *et al*., 2002; Zigman *et al*., 2005), and its role during aging has not been well‐characterized. The goal of this study was to establish the role of ghrelin during aging regarding the possible modulation of body composition and muscle function.

## Results

### Body weight, body composition, and muscle performance in young and old ghrelin WT and KO animals

**Figure 1:**
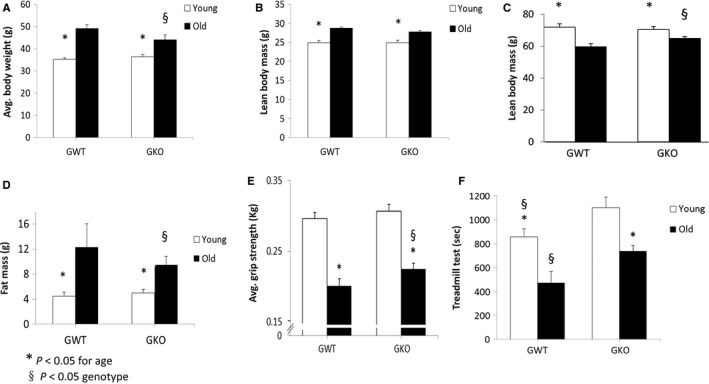
(A‐F). Body weight and composition in young and old ghrelin WT and KO animals. 6–9 months (young adult) and at 21–24 months of age (old) mice were fed standard nonsoy rodent chow (*n* = 16/group). Body weight in age‐matched animals measured in grams (A). Body composition measured by nuclear magnetic resonance (NMR), absolute lean body mass (LBM, B), and LBM percentage from body weight (C). Fat mass (D). Grip strength measured by rodent grip dynamometer (E). Treadmill activity measured in seconds where animals ran to exhaustion with increasing speed every 2 min (F). *P *<* *0.05. *young vs. old, §*P* < 0.05 ghrelin wild‐type (GWT) vs. knockout (GKO).

To address whether ghrelin deletion has an effect on body weight, body composition, and muscle mass and performance, we evaluated ghrelin wild‐type (GWT) and ghrelin knockout (GKO) mice at 6–9 months (young adult) and at 21–24 months of age (old). Older animals had higher body weight than younger animals in both genotypes (Fig. 1). Although there was no difference in body weight between young GWT and GKO animals, old GKO animals had significantly lower body weight compared to GWT. Body composition was examined by nuclear magnetic resonance (NMR). Lean body mass (LBM) increased with age in both genotypes (Fig. 1), and there were no significant differences in LBM between GWT and GKO at any age. However, the leanness (expressed as LBM percentage from total body weight) decreased with age and was partially preserved in old KO (Fig. 1). The increase in fat mass seen with aging in WT mice was also partially prevented by ghrelin deletion (Fig. 1). There were no significant differences on wet weights of hindleg muscles, and epididymal fat pads were significantly larger in old WT than in old KO mice (Table S1). To determine whether ghrelin deletion affected muscle performance, grip strength and treadmill endurance were measured. Grip strength significantly decreased with age in WT mice, and this was partially prevented by ghrelin deletion (Fig. 1). The decrease in endurance seen in older WT mice was also partially prevented by ghrelin deletion. Younger KO animals had increased endurance compared with WT animals (Fig. 1).

### Food intake, locomotor activity, and energy expenditure

**Figure 2:**
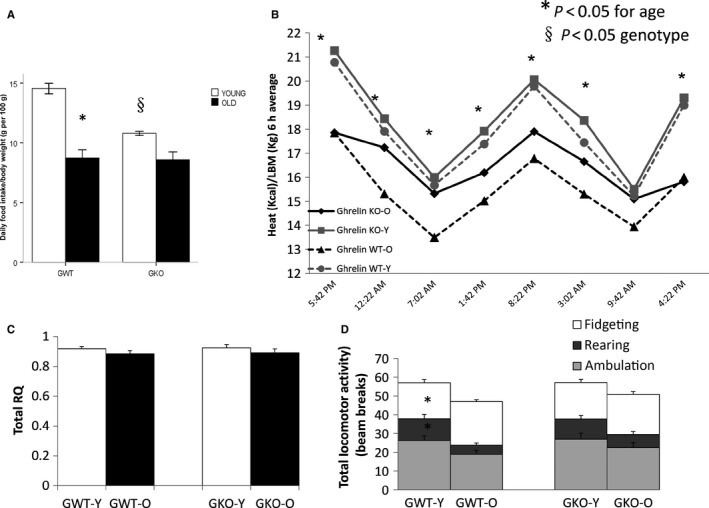
Meal pattern monitoring and calorimetry measurements. 6‐ to 9‐month‐old (young adult) and 21‐ to 24‐month‐old (old) age‐matched mice (*n* = 8/group) tested by Columbus Instruments Comprehensive Lab Animal Monitoring System (CLAMS). Daily food intake adjusted by body weight (A). Energy expenditure adjusted by LBM (B). Respiratory Quotient (RQ, C) and spontaneous total locomotor activity (D). *P *<* *0.05. *young vs. old, §*P* < 0.05 ghrelin wild‐type (GWT) vs. knockout (GKO).

Food intake in young WT mice was significantly higher than in KO animals, and it decreased significantly with age in both genotypes (Fig. 2). Differences between groups on meal size, duration, and number did not reach statistical significance (not shown). Energy expenditure (EE) adjusted by LBM decreased with age although the difference was greater and only reached significance in WT animals (Fig. 2). Respiratory quotient (RQ) obtained from indirect calorimetry was similar between young and old animals of both genotypes (Fig. 2). Spontaneous locomotor activity assessed by activity monitoring measured by interruptions of beam breakage shows that older mice are less active than younger mice in both genotypes, although the difference only reached significance in WT animals. All components of locomotor activity (fidgeting, rearing, and ambulation) as well as their sum were affected in a similar manner (Fig. 2).

### Muscle fiber type changes during aging

**Figure 3:**
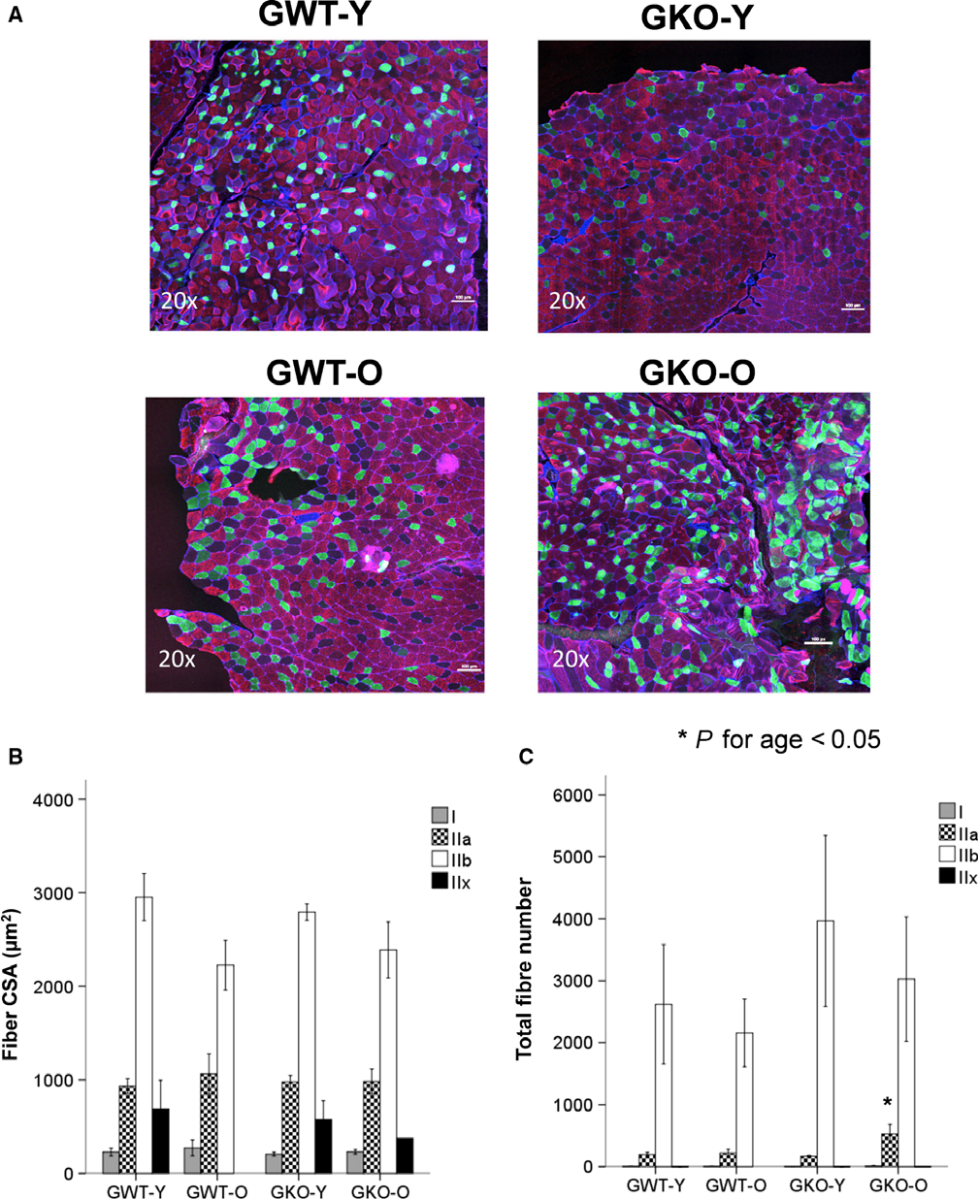
Fiber type muscle fiber distribution in young and old ghrelin WT and KO mice. Myosin heavy‐chain (MHC) immunofluorescence fiber staining (*n* = 4/group) on quadriceps muscle for type I fibers (blue), IIa fibers (oxidative, fatigue resistant, green), IIb fibers (glycolytic, red), and IIx fibers (black, A). Total fiber area distribution by fiber type: Ix, IIa, IIb, and IIx (μm^2^) (B). Total fiber number by fiber type: Ix, IIa, IIb, and IIx (C). *P *<* *0.05. *young vs. old.

Muscle fiber type distribution is altered by aging and is thought to be one of the determinants of muscle dysfunction in the elderly (Purves‐Smith *et al*., 2014). Hence, we examined whether the lack of ghrelin affected fiber type in addition to fiber distribution in quadriceps muscle in the setting of aging by immunostaining, based on myosin heavy‐chain differentiation (Bergmeister *et al*., 2016). Although there was no difference in muscle fiber cross‐sectional area (CSA) between groups, aging was associated with an increase in the number of type IIa fibers in KO animals (Fig. 3).

### AMPK‐dependent pathway activation in muscle

**Figure 4:**
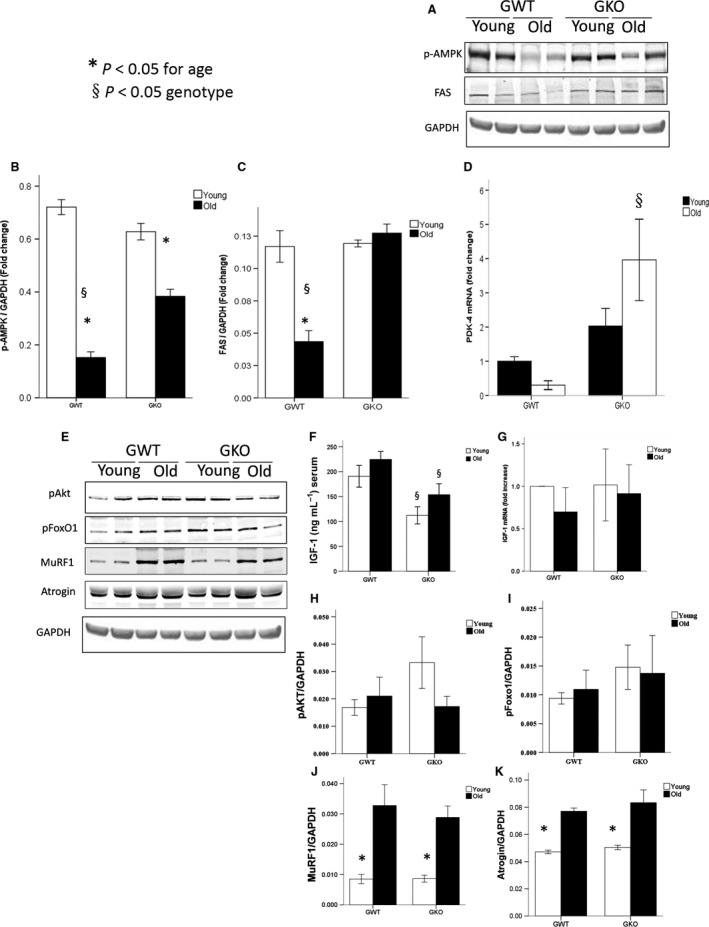
AMPK, IGF‐1, and downstream mediator protein and transcript level expression in muscles from young and old ghrelin WT and KO mice. Representative Western blots probed for p‐AMPK and FAS protein levels (A). Protein densitometry quantification for pAMPK (B) and FAS (C). Transcript level expression by RT‐qPCR for pdk‐4 (D). Representative Western blots probed for p‐AKT, p‐FoxO‐1, MuRF‐1, and MafBx (atrogin) protein levels (E). Serum IGF‐1 levels by ELISA (F). Transcript level expression by RT‐qPCR for igf‐1Ea (G). Protein densitometry quantification for p‐AKT (H), p‐FoxO‐1 (I), MuRF‐1 (J), atrogin (K). Samples normalized to GAPDH (*n* = 4/group). *P *<* *0.05. *young vs. old, §*P* < 0.05 ghrelin wild‐type (GWT) vs. knockout (GKO).

Phosphorylated adenosine monophosphate‐activated protein kinase (pAMPK), and its downstream mediators, fatty acid synthase (FAS) protein levels, and pyruvate dehydrogenase kinase (pdk)‐4 transcript levels were measured in quadriceps muscle given that this pathway regulates endurance and muscle strength (Narkar *et al*., 2008). Phospho‐AMPK levels decreased with age in WT animals, but this was partially prevented by ghrelin deletion. The decrease in FAS and pdk‐4 seen with aging was also prevented by ghrelin deletion (Fig. 4).

### IGF1, AKT, FoxO, and other proteolytic markers

Muscle strength and performance are also modulated by the ubiquitin proteasome pathway and its upstream mediators AKT, FoxO‐1, and insulin‐like growth factor‐1 (IGF‐1) (Chen *et al*., 2015; Pilling *et al*., 2016). Circulating IGF‐1 levels were lower in KO compared to WT animals and were not affected significantly by age. However, IGF‐1 mRNA levels in muscle followed a different pattern showing a nonsignificant decrease with age in WT mice. The downstream mediators of IGF‐1 activation, AKT and FoxO‐1, tended to be higher in KO animals although the differences did not reach significance. The ubiquitin ligases atrogin1 and MuRF1 increased with age in both genotypes, although the increase in MuRF1 was slightly attenuated in the KO (Fig. 4).

### Effects of ghrelin administration in old mice

**Figure 5:**
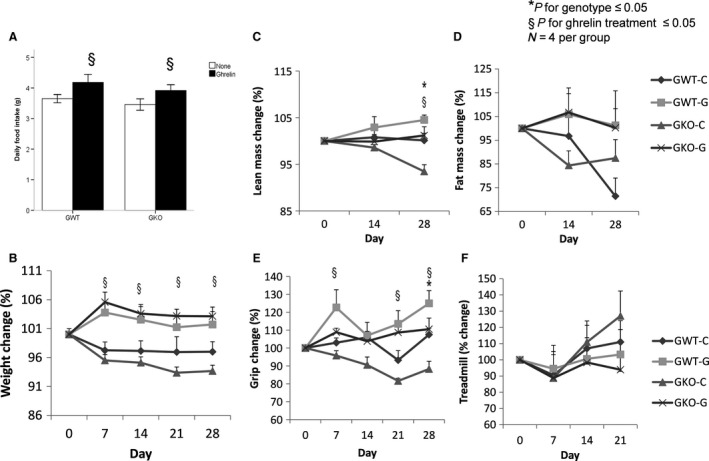
Effect of acylated ghrelin administration to old ghrelin WT and KO mice. Acylated ghrelin was administered (0.8 mg kg^−1^ twice a day, subcutaneously) for 28 days to 21‐ to 24‐month‐old, age‐matched mice (*n* = 4/group). Effects of ghrelin on daily food intake (g, A), Body weight change from baseline (%, B), Lean mass change from baseline by NMR (%, C), Fat mass change from baseline by NMR (%, D), Grip strength change from baseline (%, E), Endurance change from baseline measured by treadmill (%, F). *P *<* *0.05. *young vs. old, §*P* < 0.05 ghrelin wild‐type (GWT) vs. knockout (GKO). C: Control, G Ghrelin‐treated.

Old WT and KO animals were treated with ghrelin (0.8 mg kg^−1^ twice a day, subcutaneously) over a 28‐day period and compared to untreated animals. Ghrelin administration increased food intake and body weight in both genotypes (Fig. 5). Ghrelin also increased fat and lean body mass although the differences only reached significance at 28 days for lean body mass (Fig. 5). Ghrelin administration increased grip strength but did not have an effect on treadmill performance (Fig. 5). Ghrelin did not significantly alter muscle protein levels for phospho‐AMPK, FAS, atrogin or phospho‐AKT or transcript levels for muscle IGF‐1 and pdk4 (Fig. S1).

### Survival and inflammatory markers

**Figure 6:**
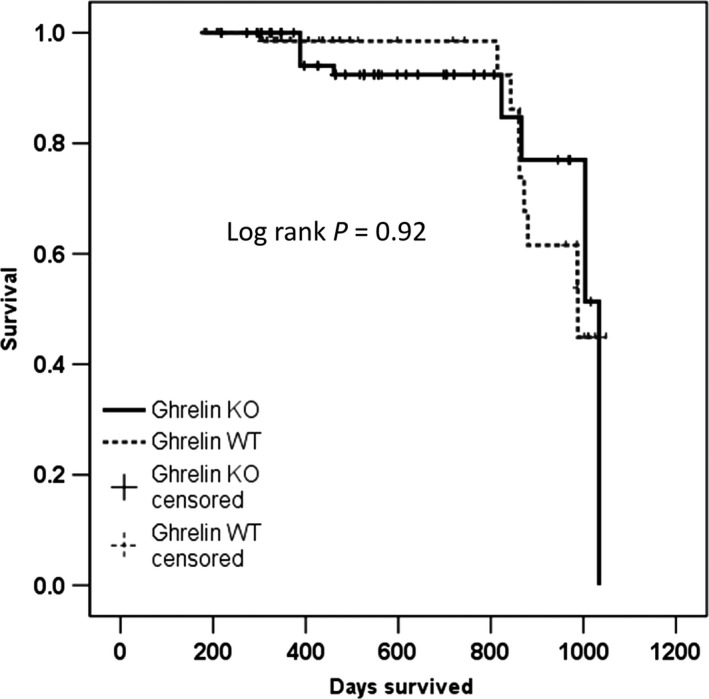
Ghrelin deletion does not affect longevity in mice. Days survived for ghrelin WT and KO mice assessed by a Kaplan–Meier curve.

Ghrelin gene deletion had no effect on survival (Fig. 6). Markers of inflammation including interleukin (IL)‐1β, 6, 10, 12p70, interferon (IFN)‐γ, and tumor necrosis factor (TNF)‐α were measured in circulation and were found to be similar between groups (Fig. S2A‐F). Knockout animals had similar litter sizes and reproduction cycles as wild‐type animals (not shown).

## Discussion

In line with what is seen in humans during aging, here we show that old wild‐type mice show an increase in body weight and fat mass, along with a significant decrease in muscle strength and endurance, in spite of muscle mass being preserved as shown previously by Van Dijk *et al*. (2017). This is not surprising because, although obesity seems to prevent the decline in muscle mass seen with aging, it is paradoxically associated with greater muscle function decline in humans (Villareal & Holloszy, 2004). Although ghrelin deletion in young animals on regular diet was previously shown not to have a significant effect on food intake, energy expenditure, or body weight (Pfluger *et al*., 2008; Sun *et al*., 2003), we show for the first time that ghrelin deletion significantly prevented body weight and fat mass gain in older mice while maintaining lean mass and muscle function when compared to wild‐type age‐matched animals.

As body weight gain develops as a result of energy imbalance, food intake and energy expenditure were studied in detail. Aging was associated with a decline in food intake, but also in spontaneous locomotor activity and total energy expenditure. Ghrelin deletion decreased food intake in young animals and partially prevented the decrease in energy expenditure seen with aging in WT mice. Given that the decrease in locomotor activity seen with aging was similar in WT and KO mice, we postulate that the difference in total energy expenditure between genotypes was primarily due to changes in resting energy expenditure. The data also suggest that a decrease in energy expenditure due to decrease locomotor activity and, perhaps also in resting energy expenditure, is the main variable driving the energy imbalance during aging in mice. Although the changes over a 48‐h period were relatively small, over time it is possible that these changes along with the lower food intake seen in young animals would be sufficient to prevent the development of obesity.

We found no differences in muscle mass or whole body lean mass between genotypes. Nevertheless, the decline in endurance and grip strength seen with aging in WT mice was also partially prevented by ghrelin deletion. Muscle quality or strength‐to‐mass ratio declines with aging in humans (Fabbri *et al*., 2017) and in rodents with muscle weakness preceding the decrease in muscle mass (Lushaj *et al*., 2008). Our data suggest that lifelong ghrelin deletion preserves muscle quality in aging mice. Human muscle changes during aging include atrophy of muscle fibers and muscle fiber loss (Ballak *et al*., 2014), but the effects of aging on muscle in c57bl/6 mice are incompletely understood (Hamrick *et al*., 2006; Somerville *et al*., 2004; van Dijk *et al*., 2017). Recent studies suggest that there is a modest amount of muscle fiber loss and no obvious decrease in cross‐sectional area (Sheard & Anderson, 2012), which is consistent with our findings. Obesity is associated with an increase in type II fiber CSA in the elderly (Gueugneau *et al*., 2015), and this could explain the phenotype in our model where there is dissociation between muscle mass and function given that mice quadriceps and gastrocnemius muscles have predominantly type II fibers (Klover *et al*., 2009). Further studies including models of obesity during aging would be needed to confirm this hypothesis.

In this study, we also show a significant increase in type IIa (fatigue resistant, more oxidative) fiber content with aging in KO compared to WT mice that is likely to be responsible for the increased endurance seen in KO aged animals. Exercise training triggers a fiber type switching causing muscles to become more oxidative (Pette & Staron, 2000). However, it is unlikely that this would cause the fiber type difference seen in our model given that spontaneous locomotor activity was not significantly different between genotypes at the ages investigated. Also, we postulate that this increase in type IIa, fatigue‐resistant, oxidative fibers could have contributed to the increased energy expenditure and subsequently decreased fat mass seen in aged KO mice as skeletal muscle fibers are major contributors to resting energy expenditure (Zurlo *et al*., 1990).

At the molecular level, the age‐related decreases in endurance and muscle strength were associated with downregulation of phospho‐AMPK and its downstream mediators FAS and *pdk4* in WT animals. These changes were partially prevented by ghrelin deletion. Previous studies have shown the importance of the AMPK pathway on improving endurance (Narkar *et al*., 2008), and this finding suggests that AMPK modulation by ghrelin could contribute to the phenotype seen in our model of increased endurance and muscle strength. The interplay between ghrelin and AMPK is not well‐understood. Acutely, ghrelin administration has been shown to decrease p‐AMPK expression in the liver but to have no effect on muscle (Barazzoni *et al*., 2005). More recently, ghrelin was shown to activate AMPK in cardiac muscle at very high doses in one report (Yuan *et al*., 2016) and to decrease AMPK phosphorylation in myoblasts in another (Han *et al*., 2015). There are no previous reports of chronic effects of ghrelin or ghrelin blockade on AMPK activation; however, it is known that AMPK target genes are key to mitochondrial biogenesis, fatty acid oxidation, and energy expenditure (Xue *et al*., 2009). Taken together, the data are consistent with the hypothesis that AMPK modulation by ghrelin may contribute to ghrelin's effects on muscle function, fat accumulation, and energy expenditure.

Given that ghrelin stimulates GH secretion and that this pathway has been postulated as a contributor to ghrelin's anabolic effects in other settings (Garcia & Polvino, 2009), we investigated the effects of ghrelin deletion on IGF‐1, GH's main effector in muscle, and on other mediators of the proteolytic pathway downstream of IGF‐1. Circulating IGF‐1 levels were decreased in ghrelin KO animals and were not significantly affected by aging. However, there was no difference in IGF‐1 transcript levels in muscle. Activation of IGF‐1 pathway leads to phosphorylation of AKT and FoxO that, in turn, prevents activation of the ubiquitin ligases atrogin and MuRF1 and decreases proteolysis via the ubiquitin proteasome system (Bodine *et al*., 2001). In our model, aging increased MAFbx (atrogin) and MuRF1 levels in spite of not changing phosphorylation levels for AKT or FoxO significantly. Ghrelin deletion did not have a significant effect on AKT, FoxO, or the ubiquitin ligases. Taken together, the data suggest that the decrease in circulating IGF‐1 levels does not translate in a downregulation of this pathway in skeletal muscle. Given that activation of this pathway is considered one of the main determinants of muscle mass, it is not surprising that ghrelin WT and KO animals showed no difference in muscle mass in spite of the differences in strength and endurance. Other pathways known to play a role in muscle metabolism such as mitochondrial biogenesis, autophagy‐lysosome system, and a central nervous system input to muscle were not studied here and should be the focus of future studies. Also, whether these effects are muscle or fiber type‐specific was not studied here.

Some studies have shown that in the setting of advanced age, there is an increased inflammatory response that is thought to contribute to the decrease in muscle performance (Roubenoff, 2004); however, other studies have not found this association (Beharka *et al*., 2001). Ghrelin has been shown to have anti‐inflammatory effects in several models including muscle wasting associated with chronic conditions such as cancer and renal failure (Chen *et al*., 2015; Deboer *et al*., 2008), and this decrease in inflammation has been proposed as a potential mechanism for its beneficial effects on muscle mass and strength. In our study, neither aging nor ghrelin deletion induced detectable changes in circulating levels of several inflammatory markers, suggesting that ghrelin's effects in this setting are not mediated by its anti‐inflammatory properties or that tissue measurements of these markers may be needed to establish such an effect.

To further examine the role of ghrelin during aging, we treated old WT and KO animals with acylated ghrelin using a regimen we have previously found to increase food intake, body weight, fat mass and muscle mass, and strength in the setting of chemotherapy and tumor‐induced cachexia (Chen *et al*., 2015; Garcia *et al*., 2008). As expected, ghrelin increased food intake and body weight in both WT and KO animals due to an increase in both lean and fat mass. Ghrelin treatment also increased grip strength and had no effect on treadmill performance in spite of ghrelin deletion having prevented the decrease in these outcomes. At the molecular level, ghrelin treatment had no sizable effect on p‐AMPK, FAS, pdk4, p‐AKT, atrogin, or IGF‐1 levels. Taken together, it is possible that ghrelin administration improves muscle strength acutely but that the long‐term effects of ghrelin on muscle are outweighed by its effects on adiposity. This could explain why conditions like cancer cachexia, where fat loss and anorexia contribute to a rapid decrease in muscle function, may improve with ghrelin administration while other more chronic conditions where food intake and fat mass are preserved such as sarcopenic obesity benefit more from long‐term ghrelin deletion. As the improvements in treadmill performance seen in old KO animals were associated with increases in the key mediator of endurance phospho‐AMPK, and muscle AMPK levels are downregulated by increased adiposity and food intake (Kahn *et al*., 2005; Steinberg *et al*., 2006), we postulate that the lack of effect of ghrelin administration on AMPK and on endurance may be due to the concomitant increases in food intake and fat mass induced by ghrelin. Further studies will be needed to test these hypotheses.

Decreased activation of the GH/IGF1 axis and leanness are thought to contribute to increased longevity in mice (Brown‐Borg *et al*., 1996; Weindruch & Walford, 1982). Hence, it was interesting to see that, in spite of having a decrease in fat mass and circulating IGF‐1 levels, survival was not affected by ghrelin deletion. It is possible that a more profound downregulation of the GH/IGF‐1 pathway than the one induced by ghrelin deletion is needed to see an effect on longevity, although involvement of other non‐GH related pathways cannot be excluded based on our model.

This study raises some important questions. Experiments conducted with ghrelin receptor knockout mice (Ghsr^−/−^) suggest that ghrelin and its effects on the release of GH and stimulation on appetite are mediated through GHSR‐1a (Sun *et al*., 2004). However, there is mounting evidence now that ghrelin also exerts direct effects on adipose tissue and skeletal muscle that are independent of this receptor (Chen *et al*., 2015; Kos *et al*., 2009; Porporato *et al*., 2013). Whether the current phenotype is mediated through GHSR‐1a receptor is not known and could not be investigated in our model. Although 70% of circulating ghrelin is derived from the stomach, ghrelin is produced locally in most tissues including skeletal muscle and adipose tissue (Ghelardoni *et al*., 2006; Gnanapavan *et al*., 2002). However, the only known ghrelin receptor (GHSR‐1a) is not expressed in muscle or fat (Gnanapavan *et al*., 2002; Sun *et al*., 2007). Given that our animals had the ghrelin gene deleted globally, it is uncertain at this point whether these effects are due to locally produced ghrelin or the result of the interplay between different tissues. The development of muscle‐specific ghrelin‐deleted animals would be needed to answer this question. Also, whether longer treatment or higher doses of exogenous ghrelin could replicate the phenotype seen in our WT when compared to KO animals, remains to be determined. The older animals in this study are comparable in terms of lifespan to ~70‐year‐old humans. The results of this study are consistent with the human literature suggesting that these animals have not yet reached the age that they lose lean mass. As these animals continue to age, it is possible that they would lose lean mass and fat mass, which may implicate ghrelin in different ways.

In summary, we show here that during aging several metabolic changes ensue including a decrease in energy expenditure and locomotor activity that, in spite of a reduction in food intake, are likely to contribute to an increase in body weight and body fat. AMPK activation in muscle is decreased during aging, leading to a decrease in oxidative capacity, muscle strength, and endurance. Lifelong deletion of ghrelin prevents the development of obesity and muscle function decline associated with normal aging. These changes were associated with a decrease in food intake, increases in AMPK phosphorylation and in type IIa (fatigue resistant, oxidative) muscle fibers and endurance. Treatment of old mice with pharmacologic doses of ghrelin increased food intake, body weight, and grip strength. These results highlight the relevance of ghrelin during aging and identify a novel mechanism for ghrelin action in muscle: an increase in AMPK phosphorylation and in type IIa muscle fibers during aging. The relevance of these findings in humans could potentially be important as ghrelin receptor agonists and antagonists are currently in clinical trials. These results may help researchers in selecting the clinical settings where these interventions are most likely to be helpful (i.e., anorexia and cachexia syndromes, where an increase in fat mass is likely to be beneficial), and the outcomes to be measured (i.e., muscle fiber type, muscle strength, appetite, and energy expenditure).

## Experimental procedures

All experiments were conducted on adult male C57BL/6J WT, *ghrelin* ^*+/+*^, and KO, *ghrelin* ^*−/−*^ mice obtained from Dr. Roy G. Smith Ph.D's laboratory (Sun *et al*., 2003). Animals were bred in‐house, individually housed (except for the survival study where animals were housed in groups of 2–4), acclimated to their cages and human handling for 7 days before the experiments were initiated and maintained on a 12/12 light/dark cycle (lights on at 6 AM). Cages were enriched with Enviropack^®^ (Lab Supply Inc., Fort Worth, TX, USA). Animals were fed regular diet (Advanced Protocol PicoLab Cat#: 3002906‐203, 5V5R). All animal procedures were approved by the Institutional Animal Care and Use Committee at Baylor College of Medicine. Experimental animal numbers ranged from 8 to 16 mice per experiment. Acylated rodent ghrelin (Anaspec, Fremont, CA, USA) or 0.9% NaCl (vehicle) was used for a ghrelin injection study. The dose of ghrelin was 0.8 mg kg^−1^ twice daily IP at 8 AM and 5 PM for 28 days. Animals were weighed daily on a scale to determine the dose of ghrelin needed.

### Body composition analysis

Fat and lean body mass were measured by nuclear magnetic resonance (NMR) as previously described with a minispec mq NMR spectrometer (Bruker optics, The Woodlands, TX, USA) (Sun *et al*., 2004). The fat represents total fat, independent of where it is localized. The intensities of the fat, muscle, and free fluid were calculated automatically from the time domain [^1^H] NMR signals by the instrument software and are expressed in grams.

### Indirect calorimetry, feeding, and activity/locomotion monitoring

Comprehensive Lab Animal Monitoring System (CLAMS, Columbus Instruments, Columbus, OH, USA) was used to assess energy expenditure, food intake, and locomotor activity. Briefly, mice were individually housed 3 days before the start of the experiment. They were then placed in the Feeding Mass Monitor which is designed to measure food consumed, frequency of consumption, and duration of each meal. The food is in powder form and is placed on a feeding assembly that rests on a precision‐modified Mettler Toledo Balance (0.01–210 g accuracy range). The mouse accesses food by eating from a spring loaded dish through an antiforaging guard; this guard also prevents full body access to the dish. Spillage is accounted for by a larger dish, beneath the main food cup, that catches anything that spills over the edge. This spillage collection cup also rests on the balance, and although spilled food is not accessible to the mouse, the mass remains on the balance. The mass of food consumed is monitored continuously. Feeding bout information is gathered by disturbances on the balance. After allowing the mice to get adjusted to the feeding mechanism for 4 days, they are then transferred to the calorimetry system. The feeding mechanism is the same as the Feeding Mass Monitor with powdered food. The mice remain in the calorimetry for 3 days after which they are removed. The calorimetry system monitors oxygen and carbon dioxide concentrations by volume at the inlet and outlet ports of a chamber through which a known flow of air is forcibly ventilated. The differences in gas concentrations along with flow information are employed in the calculations of oxygen consumption, carbon dioxide production, and respiratory exchange ratio. Along with measuring the oxygen and carbon dioxide concentrations, the locomotor activity of the mice is measured in the cages. This is performed using an Opto M3, which is a multichannel activity monitoring system for the continuous measurement of ambulatory and total movement. Infrared beams are projected at 0.5” spacing across the cage. During movement, the infrared beam is broken by the mouse, sending a signal to a central computer. The beams can detect movement along both the X and Z axes. The duration selected for quantification was derived by excluding the first 24 h and the last 24 h of the total 96 h of animal monitoring. The animals were allowed to acclimate to the monitoring chambers for 72 h prior to data monitoring.

### Cytokine assay

The measurements of circulating levels of inflammatory cytokines were performed at the MEDVAMC Division of Endocrinology laboratory, using commercially available electro‐chemiluminescence assay kits from Mesoscale (Gaithersburg, MD, USA) and according to the manufacturers’ instructions. The intra‐assay coefficient of variance (CV) was 10% or less for all assays; and the sensitivities were 0.58 pg mL^−1^, 0.77 pg mL^−1^, 0.8 pg mL^−1^, 0.18 pg mL^−1^, 0.57 pg mL^−1^, 0.28 pg mL^−1^, for IL‐1β, IL‐12p70, IFN‐γ, IL‐6, IL‐10, and TNF‐α, respectively.

### IGF‐1 assay

Circulating IGF‐1 levels were measured at the VUMC Hormone Assay and Analytical Services Core by immunoassay using a commercial kit from Millipore (St Charles, MO, USA) following manufacturer's instructions. All samples were run on the same assay, with a sensitivity of 3.2 pg mL^−1^ and intra‐assay CV < 5%. Specific steps are detailed in immunoassay procedure Milliplex Rat/Mouse IGF‐1 kit #RMIGF187F.

### Muscle preparation

Animals were sacrificed using carbon dioxide after a 6‐h fast. Muscles on the right side of the animal including quadriceps, gastrocnemius, soleus, and tibialis anterior were excised, weighed and slowly frozen with isopentane and mounted in OCT. The left side muscles were excised and frozen in liquid nitrogen and stored at −80 °C until Western blot or PCR.

### Grip strength

Mice forelimb strength was tested using a grip strength dynamometer (Columbus Instruments). The mice were placed on a metal mess platform apparatus forward facing, securing them from the base of their tail. The animals were allowed to acclimate for 30 s. The animals were then slowly pulled away with even pressure by the tail, in a motion parallel to the wire mesh. This was repeated two more times, and the highest reading was recorded.

### Treadmill

Mice perform a treadmill endurance test 3 times over the course of 5 days using the Exer‐6 m treadmill (Columbus Instruments). The treadmill is at a 10 degree incline, and the animals start running at a speed of 6 m min^−1^ and the speed is increased by 2 m min^−1^ every 2 min until the point of exhaustion (when an animal sat on the shock grid for longer than 5 s).

### Western blot analysis

Briefly, fresh frozen quadriceps muscle tissue (0.4 g) was placed in Eppendorf safe‐lock tubes with ½ volume of 0.5‐mm zirconium oxide beads (ZROB05) and 2 volumes of Cell lytic Mammalian buffer (Sigma, St. Louis, MO, USA) supplemented with PhosSTOP and Complete mini (Roche, Indianapolis, IN, USA) and homogenized with a Bullet Blender (Next Advance Inc., Averill Park, NY, USA) for 3 min. The protein concentration was measured using Pierce BCA Protein Assay (Thermo Scientific, Waltham, MA, USA). We separated protein extracts on 4–12% mini protease gels (BIO‐RAD, Hercules, CA, USA) and blotted them onto nitrocellulose membrane (BIO‐RAD). Membranes were blocked at room temperature for 1 h in 5% BSA and incubated in primary antibodies overnight at 4 °C. IR‐Dye 680/800 anti‐rabbit/mouse/Goat IgG secondary antibodies were used (LI‐COR, Lincoln, NE, USA) for 1 h at room temperature. After three washes in TBS‐T, we scanned the blots with the LI‐COR Odyssey (LI‐COR) and quantified them with Image‐Pro plus on the basis of direct fluorescence measurement. The following antibodies were used: *p*‐Akt (Ser 437) (Cell Signaling, Danvers, MA, USA) *p‐* FoxO‐1 (T32) (Cell Signaling), *p‐*AMPK (T172) (Cell Signaling), FAS (Sigma‐Aldrich, St. Louis, MO, USA), atrogin (FBX032) (ThermoFisher, Waltham, MA, USA), muscle‐specific RING finger protein (MuRF1) (Santa Cruz Biotechnology, INC. Dallas, TX, USA), and glyceraldehyde‐3‐phosphate dehydrogenase (GADPH) from Cell Signaling. Normalization for p‐AKT and p‐AMPK was only performed to GAPDH to optimize the use of the limited amount of protein available for Western blotting in our experiments.

### Muscle fiber staining

10‐μm‐thick frozen muscle fiber sections were allowed to stand at room temperature prior to treatment with cold acetone for 15 min. Slides were then washed in phosphate‐buffered solution (PBS) for 10 min and then incubated in 0.5% Triton X‐100/PBS for 15 min followed by PBS washing. Slides were dried and treated with Image‐iT FX signal enhancer (Molecular Probes, Grand Island, NY, USA) and incubated in AffiniPure Goat anti‐Mouse IgG, Fab fragment (Jackson ImmunoResearch, West Grove, PA, USA) in 4% BSA IgG free (Jackson ImmunoResearch) for 1 h. Blocking was accomplished with 5% NGS in 4% BSA for 30 min. Slides were then incubated with myosin heavy‐chain primary antibodies: BA‐D5, alpha and beta (IgG2b 1:50), SC‐71, IIA (IgG1 1:500), BF‐F3, IIB (IgM 1:50) Developmental Studies Hybridoma Bank (Iowa City, IW), and antilaminin (Sigma‐Aldrich) (1:250) overnight at 4 °C in 4% BSA. Following overnight incubation, slides were washed with PBS. Secondary antibodies: AF546 Goat anti‐mouse IgM, AF488 Goat anti‐mouse IgG1, AF647 Goat anti‐mouse IgG2b, AF405 Goat anti‐rabbit (1:500) (Life Technologies, Grand Island, NY, USA) in 4% BSA at room temperature. Slides were then washed in PBS before mounted with Slow Fade Gold (Molecular Probe, Grand Island, NY) followed by coverslip. Fiber quantification was completed by ImageJ software was used for cross‐sectional area quantification. Rasband, W.S., ImageJ, U. S. National Institutes of Health, Bethesda, Maryland, USA, http://imagej.nih.gov/ij/, 1997–2014.

### Assay of mRNA expression real‐time PCR

Total RNA was isolated from quadriceps tissues (40–60 mg), using the guanidinium method (Trizol; Invitrogen, Carlsbad, CA, USA). Transcript levels were measured by real‐time PCR (9700HT Sequence Detection System; Applied Biosystems, Foster City, CA, USA). 500 ng of total RNA was reverse transcribed (QuantiTect Reverse Transcription Kit, Qiagen, Germantown, MD USA) to cDNA. Primers and probes for real‐time PCR amplification were selected using Primer Express Software (Applied Biosystems, Table S2 **).** The probe for target genes was labeled at the 5_ end with a reported dye FAM (6_‐carboxyfluorescein) and at the 3_ end with a quencher dye TAMRA (6_‐carboxytetramethylrhodamine). The reporter and quencher dyes are in close proximity on the probe, resulting in suppression of reporter fluorescence. The probe–exon is designed to hybridize to a specific sequence within the PCR product. The 5_ ‐ to 3_ cleave activity of the *Taq*DNApolymerase allows for separation of the reporter from close proximity of the quencher dye, resulting in fluorescence of the reporter dye. The resulting signal is measured at each amplification cycle on the ABI Sequence Detection System (Applied Biosystems), thus allowing the measurement of sample abundance in the linear phase of amplification. Target genes were amplified using aliquots of the same cDNA sample, and final quantification of each sample was achieved by coamplified relative standard curve.

### Statistical analysis

Data are presented in the figures as mean ± SEM (standard error of mean). The animal number per group is indicated by *n*. Significant differences between groups were evaluated by ANOVA tests using IBM spss predictive analytics software 21.0 (SPSS Inc. Chicago, IL, USA). Mice were followed until demise, and survival analysis was performed using Kaplan–Meier survival analysis. *P* values ≤ 0.05 were considered statistically significant.

## Funding

This work was funded by the U.S. Dept of Veterans Affairs (MERIT grants I01‐BX002807 and I01 CX000174), and NIH Grant AG040583 to JMG. Dr Guillory was supported by a training grant from the NIA (T32AG000183). Dr Chen and Luo are supported by National Natural Science Foundation of China (81072262, 81372944). We thank the University of Washington DERC (P30 DK017047) and NORC (P30 DK035816), Vanderbilt MMPC (supported in part by U24 DK59637), VUMC Hormone Assay and Analytical Services Core (supported by NIH grants DK059637 and DK020593), and University of Virginia (DK076037) for their help.

## Author contributions

JMG and BG designed experiments and wrote the manuscript. BG, BA, BI, TH, SP, AS, JAC, and JL conducted experiments. JMG, BG, MC, SB, AM, and YH analyzed data.

## Conflicts of interests

Authors report no conflict of interests.

## Supporting information


**Fig. S1** Effect of acylated ghrelin administration on protein and transcript level expression in muscles from old ghrelin WT and KO mice.
**Fig. S2** Inflammatory cytokines in serum.

**Table S1** Muscle and fat pad weights in mg (mean ± SD).
**Table S2** RT‐PCR primers.
