# Supplementary material for: Deletion of ghrelin prevents aging‐associated obesity and muscle dysfunction without affecting longevity
Source: Aging Cell. 2017 Jun 6;16(4):859–69. doi: 10.1111/acel.12618 (PMC5506439; doi:10.1111/acel.12618)
Supplement: Supplementary file 1 — Fig. S1 Effect of acylated ghrelin administration on protein and transcript level expression in muscles from old ghrelin WT and KO mice. Fig. S2 Inflammatory cytokines in serum. [file ACEL-16-859-s001.pptx]

## Slide 1
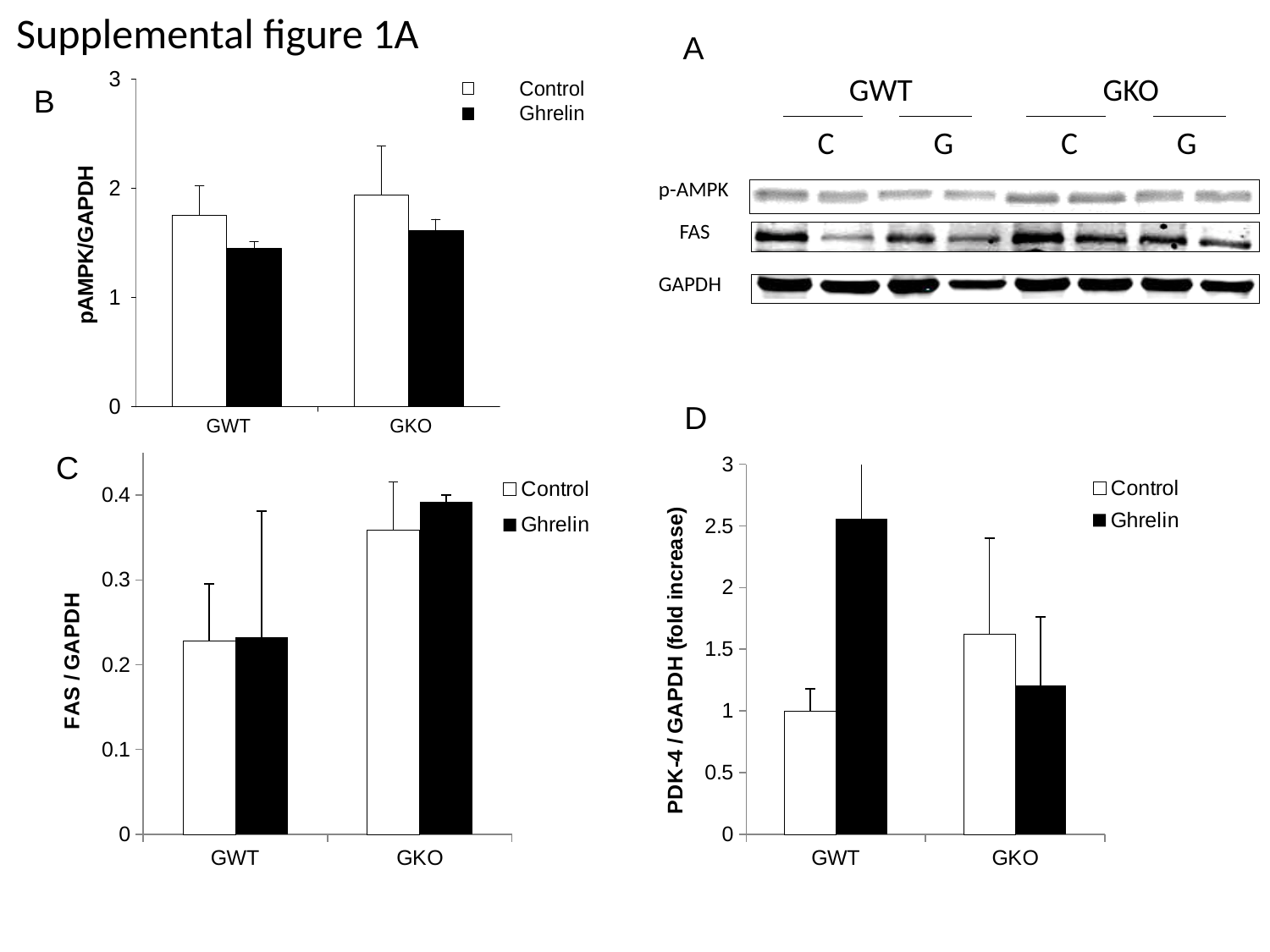

Supplemental figure 1A
A
GWT
GKO
B
C
G
C
G
p-AMPK
FAS
GAPDH
D
### Chart
| Category | Control | Ghrelin |
|---|---|---|
| GWT | 0.227983128675019 | 0.232327098155549 |
| GKO | 0.35935549171485 | 0.39230698817868 |
### Chart
| Category | Control | Ghrelin |
|---|---|---|
| GWT | 1.0 | 2.560006033360545 |
| GKO | 1.622855063075262 | 1.203623802279407 |

## Slide 2
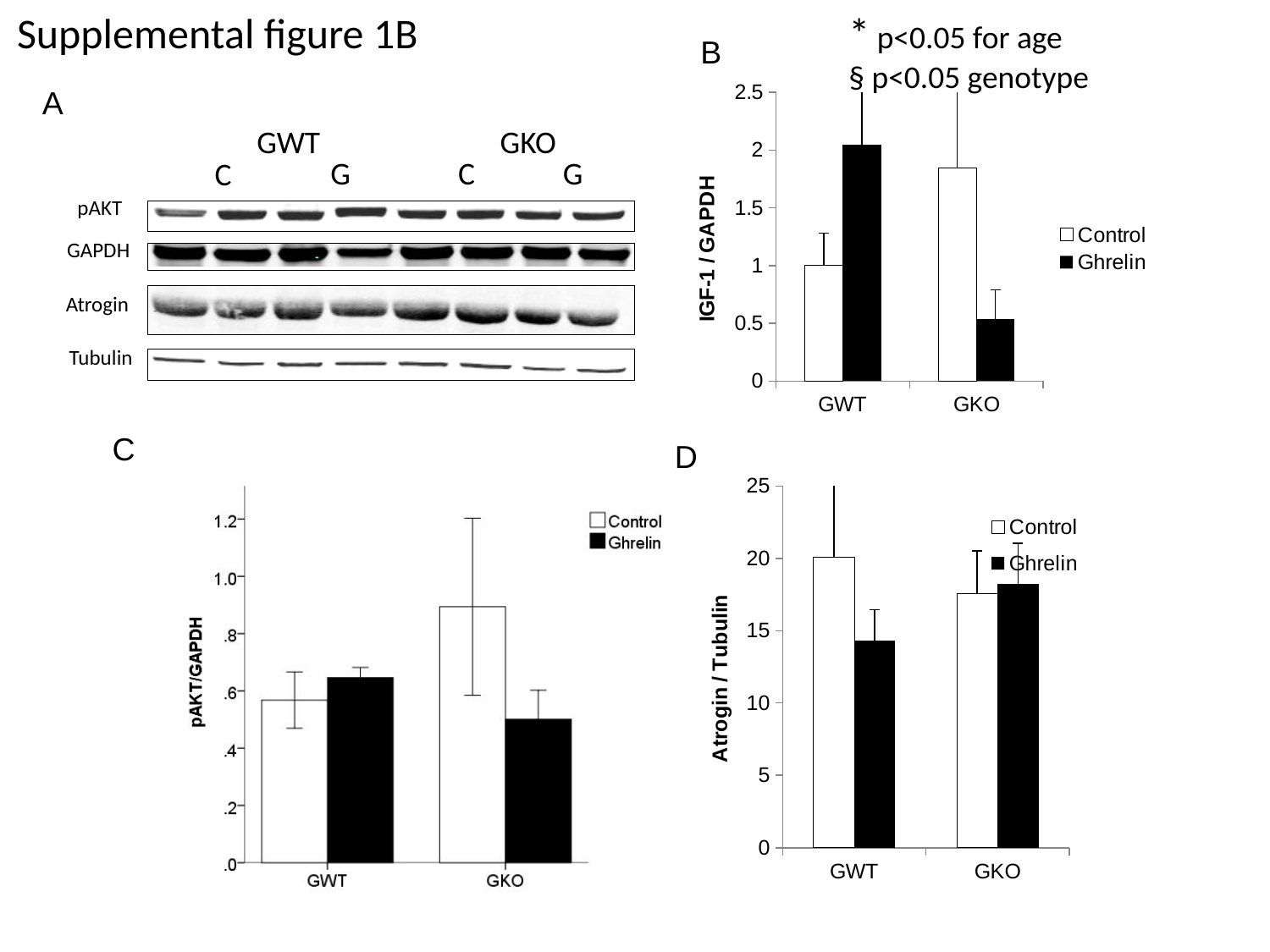

Supplemental figure 1B
* p<0.05 for age
§ p<0.05 genotype
B
### Chart
| Category | Control | Ghrelin |
|---|---|---|
| GWT | 1.0 | 2.044329390007159 |
| GKO | 1.845291108102736 | 0.536823303752798 |A
GWT
GKO
G
C
G
C
pAKT
GAPDH
Atrogin
Tubulin
C
D
### Chart
| Category | Control | Ghrelin |
|---|---|---|
| GWT | 20.10883515580882 | 14.309935511303237 |
| GKO | 17.6017316017316 | 18.222394220846233 |

## Slide 3
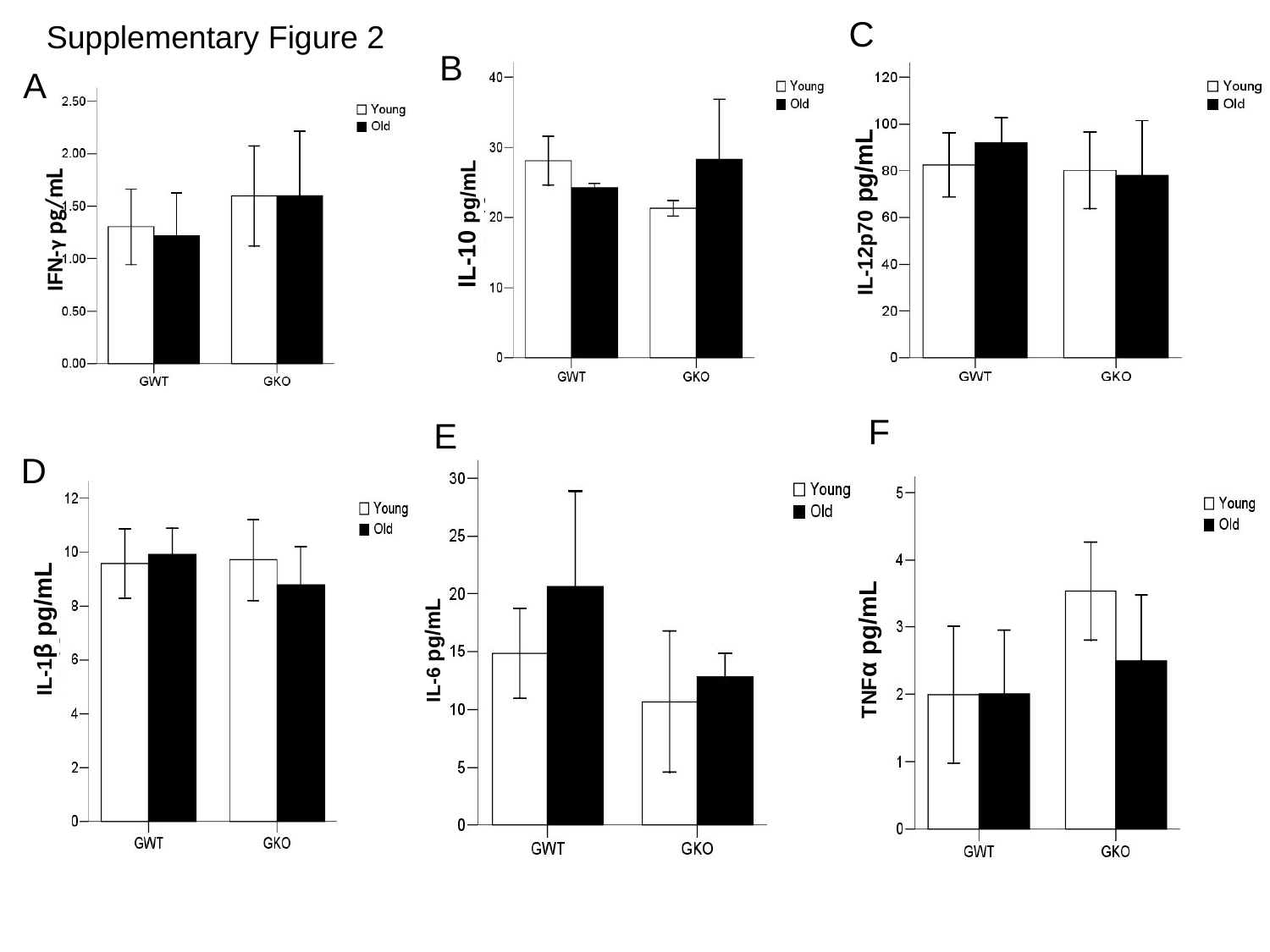

C
Supplementary Figure 2
B
IL-10 pg/mL
IL-12p70 pg/mL
A
IFN-γ pg/mL
F
E
D
IL-6 pg/mL
IL-1β pg/mL
TNFα pg/mL
