# Supplementary material for: Deletion of ghrelin prevents aging‐associated obesity and muscle dysfunction without affecting longevity
Source: Aging Cell. 2017 Jun 6;16(4):859–69. doi: 10.1111/acel.12618 (PMC5506439; doi:10.1111/acel.12618)
Supplement: Supplementary file 2 — Table S1 Muscle and fat pad weights in mg (mean ± SD). Table S2 RT‐PCR primers. [file ACEL-16-859-s002.docx]

**Supplemental Figures Legends**

**Supplemental Figure 1A.** **Effect of acylated ghrelin administration on protein and transcript level expression in muscles from old ghrelin WT and KO mice. Related to figure 6.** Representative western blots probed for p-AMPK and FAS protein levels (A). Protein densitometry quantification for pAMPK (B) and FAS (C). Transcript level expression by RT-qPCR for pdk-4 (D). Samples normalized to GAPDH (n=4/group). *P* < 0.05. * young vs. old, § p<0.05 ghrelin wild-type (GWT) vs. knock-out (GKO). C: Control, G Ghrelin-treated.

**Supplemental Figure 1B.** **Effect of acylated ghrelin administration on protein and transcript level expression in muscles from old ghrelin WT and KO mice. Related to figure 6.** Representative western blots probed for p-AKT and MafBx (atrogin) protein levels (A). Transcript level expression by RT-qPCR for igf-1 (B). Protein densitometry quantification for p-AKT (C) and Atrogin (D). Samples normalized to GAPDH (n=4/group). *P* < 0.05. * young vs. old, § p<0.05 ghrelin wild-type (GWT) vs. knock-out (GKO). C: Control, G Ghrelin-treated.

**Supplemental Figure 2.** **Inflammatory cytokines in serum. Related to figure 7.** Serum cytokine levels measured by electro-chemiluminescence assays for: INFƳ (A), IL-10 (B), IL-12p70 (C), IL-1B (D), IL-6 (E) and TNFα (F).

**Supplemental Table 1. Muscle and Fat Pad Weights in mg (mean±SD)**

|  | **GWT-Young** | **GWT-Old** | **GKO-Young** | **GKO-Old** |
| --- | --- | --- | --- | --- |
| **Quadriceps** | **21.3±2.7** | **17.6±1.3** | **20.5±1.3** | **18.6±0.6** |
| **Gastrocnemius** | **13.8±0.5** | **16±0.4** | **15.9±0.6** | **14.4±1.2** |
| **Soleus** | **0.7±0.2** | **1.6±0.2** | **1.8±1.1** | **1.3±0.5** |
| **TA** | **5±1.3** | **7.1±0.2** | **5±0.3** | **5.9±0.6** |
| **EDL** | **0.5±0.1** | **1.4±0.1** | **1±0.1** | **0.8±0.1** |
| **Epididymal fat** | **80.8±8.8** | **162±39** | **67.5±5.8** | **55±7.1**§ |
| **Inguinal fat** | **28.6±2** | **96±33.5** | **33.2±12.4** | **20.7±3.5** |

EDL: Extensor digitorum longus, TA: Tibialis Anterioris. *P* < 0.05. * young vs. old, § p<0.05 ghrelin wild-type (GWT) vs. knock-out (GKO). C: Control, G Ghrelin-treated.

**Supplemental Table 2. RT-PCR primers**

| mRNA | RT-PCR primers | |
| --- | --- | --- |
|  | Forward | Reverse |
| GAPDH | 5′-ACCACCATGGAGAAGGCCGG-3′ | 5′-CTCAGTGTAGCCCAAGATGC-3′ |
| Igf-1Ea | 5′-GGTGGTTTATGAATGGTT-3′ | 5′-AGGGTGTGTCTAATGGAG-3′ |
| Pdk4 | 5’-ATTTCTGACCGAGGAGGCGGTGTTC-3’ | 5’-CAGGTGTTGGAGCAGTGGAGTAC-3’ |
